# Highly Aggressive Invasive Race Group *Pst*S2 in Russian Populations of the Wheat Yellow Rust Pathogen

**DOI:** 10.1134/S0012496623700527

**Published:** 2023-10-13

**Authors:** E. L. Shaydayuk, E. I. Gultyaeva

**Affiliations:** All-Russian Institute of Plant Protection, 196608 St. Petersburg, Russia

**Keywords:** invasive races, molecular markers, *Puccinia striiformis*, *Yr*-genes, population, *Triticum aestivum*

## Abstract

The expansion of the area of harmfulness of the wheat yellow rust pathogen (*Puccinia striiformis*) (*Pst*) has be observed all over the world in the 2000s. This is due to the emergence of new highly aggressive invasive groups of races *Pst*S1 and *Pst*S2, adopted to the high temperatures, and also as a result of virulence mutations of regional pathogen populations. SCAR-markers were developed for identification of invasive races, and pathogen collections from many countries were studied. In these studies in first in Russia, the analysis of regional populations of *P. striiformis* for the presence of invasive races *Pst*S1 and *Pst*S2 was carried out. Single pustule isolates were obtained from urediosamples collected from common and durum wheat, triticale and wild grasses in seven regions of the Russian Federation (North Caucasian, Northwestern, Central Black Earth, Lower Volga, Middle Volga, Volga–Vyatka, West Siberian) in 2019–2020. In total 82 isolates were studied. Using SCAR markers, three genotypes were identified in the studied collection of *P. striiformis*, and one of which belongs to the invasive group *Pst*S2. The other two genotypes had a different origin (other). Isolates of *Pst*S2 group were received from pathogen population samples collected in the Russian Northwest in 2020. Virulence analysis revealed two phenotypes among them: *Pst*S2_R1 (3 isolates) and *Pst*S2_R2 (1 isolate). According to information from the Global Rust Reference Center (http://www.wheatrust.org/), the main characteristic of isolates from invasive *Pst*S2 group is virulence to wheat lines with resistance genes *Yr2*, *Yr6*, *Yr7*, *Yr8*, *Yr9*, and *Yr25*. The Russian R1 phenotype *Pst*S2 was also characterized by virulence to these genes, as well as to *Yr1*, *Yr32*, and *YrSp*. The *Pst*S2_R2 phenotype differed from *Pst*S2_R1 for avirulence to *Yr25* and virulence to *Yr3* and *Yr4*. The main difference of Russian *Pst*S2 isolates with detected in other countries is virulence to wheat lines with genes *Yr4*, *Yr32*, and *YrSp*. The first detection of invasive races in the Northwest of Russia indicates the relevance of annual monitoring of regional populations of *P. striiformis.*

## INTRODUCTION

Wheat is the main strategic crop. Diseases are one of the factors reducing the yield of grain and the quality of its products [1]. Rust is the most widespread disease of this crop. There are three species of rust pathogens occurring on wheat: *Puccinia triticina* Erikss., *P. graminis* Pers., and *P. striiformis* West. [2], which are characterized by high evolutionary potential. The accelerated rate of mutations predetermines the emergence of new races. Greater migratory propensity of the pathogens favors their rapid spread throughout countries and continents [3, 4].

Brown rust had been economically significant for wheat until 2005 in most countries, including Russia [5, 6]. In the modern period, situation has changed and brown rust has been replaced by yellow and stem rusts. The yellow rust pathogen affects both cultivated and wild type cereals, including soft and hard wheat, triticale, barley and rye. The symptoms of the disease are observed on leaves, leaf sheaths, spikelet scales and, less frequently, on stems, as lemon yellow uredinio-pustules arranged in longitudinal rows [7]. Therefore, another popular name of this disease is stripe rust.

Air humidity and temperature are the major factors that determine successful development of rust fungi. *P. striiformis* develops at lower temperatures (2–15°С) and higher atmospheric humidity [4], which limited wide distribution of the pathogen, in contrast to more plastic species *P. triticina* and *P. graminis*.

Uredinio-spores of the yellow rust pathogen in the winter period die at –4°С, while fungal mycelium in living tissue of a wheat leaf remains viable even at lower temperatures [8]. J.W. Hendrix and E.H. Lloyd (1966) report that one uredinio-pustule per 1 ha of wheat is enough for epiphytotic development of the disease under favorable weather conditions in spring [9]. In case of favorable weather conditions and early development of the disease, yield losses can be up to 100% [10].

Until recently, wheat yellow rust has been one of the diseases of regional significance worldwide. In the 2000s, its area and harmfulness began to increase substantially. Epiphytotics of the disease are regularly observed in West Europe, Central and East Asia, in the Middle East, North and South Africa, North and South America, Australia, Central Asia and Kazakhstan [4, 11–17]. This is due to the appearance of new highly aggressive groups of races *Pst*S1 and *Pst*S2 that are able to develop at high temperatures. In addition, rapid mutations of the pathogen in virulence have been noted. The new races appearing in West Europe, Australia, and North America have overcome the resilience of previously resistant wheat and triticale varieties and now rapidly spread around the world [18, 19].

The *Pst*S1 race group was found for the first time in Kenia in 1982. Later, its presence was recorded in other countries of East Africa: Ethiopia (1986), Rwanda, Burundi (1988), Tanzania (1990). It was recorded for the first time in the United States in 2000 and in Australia in 2002, being the cause of the strongest epiphytotics of yellow rust in these countries [20–23]. The difference between *Pst*S1 and other globally widespread races is the adaptation to high temperatures, which is untypical of this pathogen [21, 24, 25]. At present, the *Pst*S1 group is annually detected in East Africa and, in the years of epiphytotics, in the United States and Australia (https://agro.au.dk/ forskning/internationale-platforme/wheatrust); however, it has not been found in other regions of the world.

The *Pst*S2 group is a result of mutation in *Pst*S1, i.e., in essence, is its sister line. In contrast to *Pst*S1, it has become widespread. It was observed in the Middle East and in North Africa in the 1990s; in West Europe in 2002; in West and Central Asia in 2003 [23, 26–28]. In 2015–2016, *Pst*S2 isolates were detected in the countries bordering Russia: Ukraine and Azerbaijan. It is hypothesized that the emergence and distribution of new races *Pst*S1 and *Pst*S2 can be due to overcoming the efficiency of resistance gene *Yr9* in the Middle East and in South Asia in the 1980s and 1990s [29].

The Russian populations of the yellow rust pathogen have not been studied for the presence of invasive races *Pst*S1 and *Pst*S2 to date. At the same time, both groups are characterized by the high rate of variability with respect to virulence and microsatellite loci, which determines the need for their monitoring. The present work is aimed at the molecular-genetic analysis of the Russian populations of *P. striiformis* for the presence of highly aggressive invasive groups of races *Pst*S1 and *Pst*S2.

## MATERIALS AND METHODS

The infection samples represented by the leaves with *P. striiformis* uredinio-pustules were obtained from seven regions of the Russian Federation: North Caucasian (Dagestan, Krasnodar Krai), Northwestern (Leningrad Oblast), Central Black Earth (Tambov Oblast), Lower Volga (Saratov Oblast), Middle Volga (Samara Oblast), Volga–Vyatka (Kirov Oblast), and West Siberian (Novosibirsk Oblast, Krasnoyarsk Krai) in 2019–2020. They were collected from soft and hard wheat, triticale and cereal grasses on experimental fields, state variety plots (SVP) and industrial crops. The origin of infectious material is given in Table 1.

**Table 1.** Characteristics of the studied collections of *Puccinia striiformis*

Uredinium sampling site	Year of sampling	Crop	Number of monopustule isolates
Dagestan	2020	Hard wheat	2
		Soft wheat	3
Krasnodar Krai	2019	Soft wheat	4
		Cereal grasses	5
	2020	Hard wheat	4
		Soft wheat	4
Leningrad Oblast	2019	Triticale	4
		Soft wheat	5
		Hard wheat	4
	2020	Triticale	11
		Soft wheat	17
Novosibirsk Oblast	2019	Soft wheat	4
	2020	Soft wheat	4
Kirov Oblast	2020	Soft wheat	2
Tambov Oblast	2020	Soft wheat	2
Krasnoyarsk Krai	2020	Soft wheat	4
Saratov Oblast	2020	Soft wheat	2
Samara Oblast	2020	Hard wheat	1
Total isolates			82

Monopustule isolates were obtained as described previously [30]. For molecular studies, 82 *P. striiformis* monopustule isolates were isolated and multiplied.

DNA was extracted from uredinio-spores of the *P. striiformis* monopustule isolates according to Justesen et al. (2002) [31]. Spores were destroyed using a FastPrep®-24 homogenizer.

A set of SCAR markers (SCP19M24a1, SCP19M24a2, SCP19M26a1, and SCP19M26a2) was used to identify the invasive groups of races PstS1 and PstS2 [23]. The following amplification program was used: 94°C, 3 min; 35 cycles (94°C, 30 s; 62°C, 1 min; 72°C, 30 s); 72°C, 5 min. PCR products were analyzed in 1.5% agarose gel in 1% TBE buffer.

The results were interpreted in accordance with the following principle: the isolates belonging to the PstS1 group have amplification products of all four markers: SCP19M24 a1, 485 b.p.; SCP19M24 a2, 385 b.p.; SCP19M26a1, 491 b.p.; SCP19M26a2, 262 b.p; the isolates of the PstS2 group have amplification products of the SCP19M24a1, SCP19M24a2, and SCP19M26a2 markers.

Virulence was analyzed in the *P. striiformis* isolates assigned to the defined invasive groups by the results of molecular analysis [30]. The virulence testers were Avocet (AvS NIL) lines with the genes *Yr1*, *Yr5*, *Yr6*, *Yr7*, *Yr8*, *Yr9*, *Yr10*, *Yr15*, *Yr17*, *Yr18*, *Yr24*, *Yr27*, *YrSp* and varieties Chinese 166 (*Yr1*), Lee (*Yr7*, *Yr+*), Heines Kolben (*Yr6*, *Yr+*), Vilmorin 23 (*Yr3*), Moro (*Yr10*, *YrMor*), Strubes Dickkopf (*YrSD*, *Yr+*), Suwon 92/Omar) (*YrSu*, *Yr+*), Hybrid 46 (*Yr4*, *Yr+*), Reichersberg 42 (*Yr7*, *Yr+*), Heines Peko (*Yr6*, *Yr2*), Nord Desprez (*Yr3*, *YrND*, *Yr+*), Compair (*Yr8*, *Yr19*), Carstens V (*Yr32*, *Yr+*), Spaldings Prolific (*YrSP*, *Yr+*), Heines VII (*Yr2*, *Yr+*). The type of reaction was determined by the scale of Gassner and Straib (1926) [32]. Plants were classified as resistant with points 0‒2 and as susceptible with points 3, 4 and X—to susceptible.

## RESULTS AND DISCUSSION

**Fig. 1.**
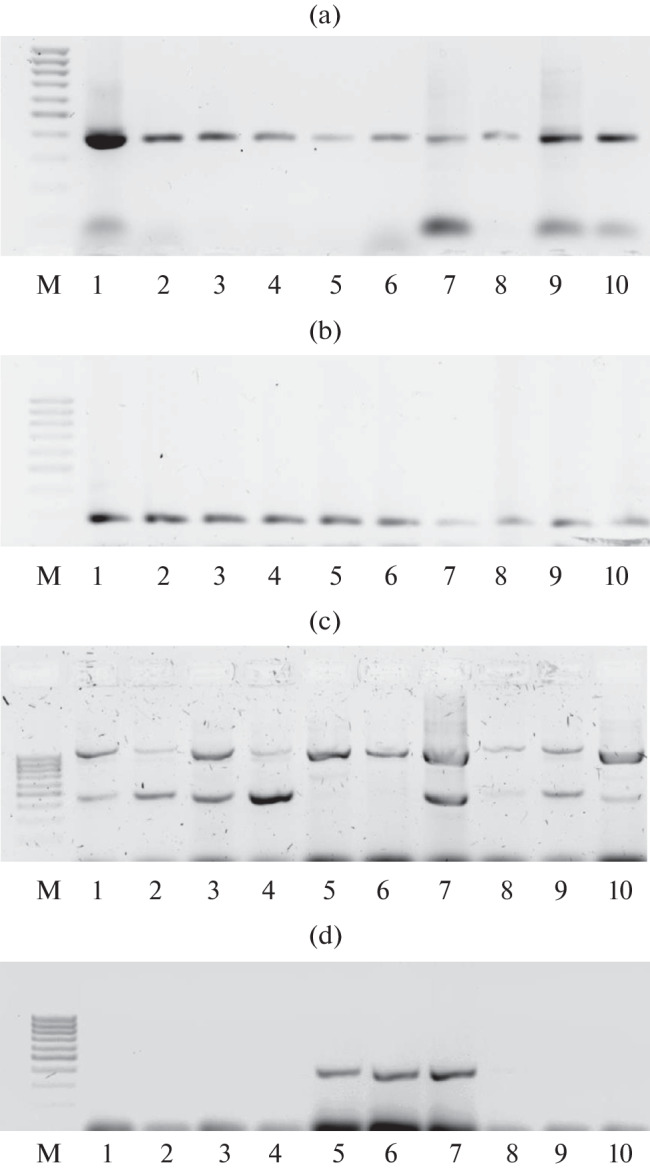
The electrophoregram of PCR for *Puccinia striiformis* isolates (nos. 1–10) with SCAR markers SCP19M24a and SCP19M26a: (a) the SCP19M24a2 marker (amplification product size 385 bp), (b) the SCP19M26a2 marker (262 bp), (c) the SCP19M24a1 marker (405 bp), (d) SCP19M26a1 (491 bp). M, marker 100 pp. DNA [manufacturer “Dialat” (http://dialat.ru/f/ m100rus.pdf )]. Isolates nos. 5, 6, 8 are representatives of the invasive PstS2 group.

Molecular analysis was carried out for 82 Russian isolates of the yellow rust pathogen of broad geographic origin. Amplification products of 385 and 262 b.p. in size, which are typical of markers SCP19M24a2 and SCP19M26a2, have been detected in all *P. striiformis* samples under study. The SCP19M26a1 marker (491 b.p.) was also amplified in most isolates, with the exception of three northwestern, one Kirov and two Krasnodar isolates from soft wheat. A 405-b.p. diagnostic fragment of the SCP19M24a1 marker was found in four northeastern isolates from soft wheat. The examples of electrophoregrams are shown in Fig. 1.

Molecular analysis has shown three genotypes in the studied collection of *P. striiformis* (Table 2). According to Walter et al. (2016), isolates having diagnostic fragments with the SCP19M24a1, SCP19M24a2, and SCP19M26a2 markers belong to the *Pst*S2 invasive group. Such *P. striiformis* isolates were detected in the samples of the Northwestern population collected in 2020 on the experimental field of Pushkin VIR Laboratories (St. Petersburg, Pushkin) (2 isolates) and Leningrad SVP (Gatchina District, Rozhdestveno and Volosovo). Two other genotypes differed from *Pst*S1 and *Pst*S2, which was indicative of their different origin. In global literature, such isolates are referred to as “other” [23]. The examples of electrophoregrams are shown in Fig. 1.

**Table 2.** Characteristics of the *Puccinia striiformis* genotypes determined using SCAR markers and their representation in the pathogen collection under study

Group	Presence of amplification product of the marker*				
	SCP19M24a1	SCP19M24a2	SCP19M26a1	SCP19M26a2	Frequency, %
1 (PstS2)	+	+	–	+	4.9
2	–	+	+	+	87.8
3	–	+	–	+	7.3

*Presence (+), absence (–) of amplification product.

Virulence analysis of the isolates belonging to the *PstS*2 group of races has shown that three isolates (two from Pushkin and one from Volosovo) are similar in the phenotype of virulence, while the Rozhdestveno isolate was different. All isolates are avirulent to the Avocet lines with the genes *Yr5*, *Yr10*, *Yr15*, *Yr17*, *Yr24*, *Yr26* and to varieties Moro (*Yr10*, *YrMor*), Nord Desprez (*Yr3*, *YrND*, *Yr+*) and are virulent to the lines with the genes *Yr1*, *Yr6*, *Yr8*, *Yr9*, *Yr27*, *YrSp* and varieties Chinese 166 (*Yr1*), Lee (*Yr7*, *Yr+*), Heines Kolben (*Yr6, Yr+*), Suwon 92/Omar) (*YrSu*, *Yr+*), Reichersberg 42 (*Yr7*, *Yr+*), Heines Peko (*Yr6*, *Yr2*), Compair (*Yr8*, *Yr19*), Carstens V (*Yr32*, *Yr+*), Spaldings Prolific (*YrSP*, *Yr+*), Heines VII (*Yr2*, *Yr+*). The differences in virulence were observed in varieties Vilmorin 23 (*Yr3*), Strubes Dickkopf (*YrSD*, *Yr+*), Hybrid 46 (*Yr4*, *Yr+*) and the Avocet line with the gene *Yr18*. The Rozhdestveno isolate was avirulent to the line with *Yr18* and variety Strubes Dickkopf and virulent to Vilmorin 23 and Hybrid 46.

The analysis of a vast collection of isolates at the Global Rust Reference Center has shown that isolates from *Pst*S1 and *Pst*S2 invasive groups have no specific differences in virulence and can be similar both to each other and to isolates not belonging to these groups [33]. Worldwide, three and nine phenotypes of virulence have been determined in the *Pst*S1 and *Pst*S2 groups, respectively (Table 3). The most typical feature of isolates from both invasive groups is virulence to the samples with the genes *Yr2*, *Yr6*, *Yr7*, *Yr8*, *Yr9*, and *Yr25* [21, 28]. The Russian phenotype *Pst*S2_R1 represented by three isolates was characterized by virulence to all of these genes and, in addition, was virulent for *Yr1*, *Yr32*, and *YrSp*. The *Pst*S2_R2 phenotype differed from *Pst*S2_R1 in avirulence to *Yr25* and virulence to *Yr3* and *Yr4*. The main difference between the Russian isolates of PstS2 group and those identified in other countries is virulence to the lines with the genes *Yr4*, *Yr32*, and *YrSp*.

**Table 3.** Virulence of the *Puccinia striiformis* isolates belonging to the *Pst*S1 and *Pst*S2 groups abroad (http://www.wheatrust.org/) and in Russia

Group	Phenotype	Virulence*	Region of maximum representation
*Pst*S1	*Pst*S1	–, 2, –, –, –, 6, 7, 8, 9, –, –, –, –, 25, –, –, –, AvS	USA, Australia
	*Pst*S1,v1	1, 2, –, –, –, 6, 7, 8, 9, –, –, –, –, 25, –, –, –, AvS, –	East Africa
	*Pst*S1,v1,v27	1, 2, –, –, –, 6, 7, 8, 9, –, –, –, –, 25, 27, –, –, AvS, –	'*'*
*Ps*tS2	*Pst*S2	–, 2, –, –, –, 6, 7, 8, 9, –, –, –, –, 25, –, –, –, AvS, –	East Africa, West and South Asia
	*Pst*S2,v1	1, 2, –, –, –, 6, 7, 8, 9, –, –, –, –, 25, –, –, –, AvS, –	East Africa
	*Pst*S2 v3	–, 2, 3, –, –, 6, 7, 8, 9, –, –, –, –, 25, –, –, –, AvS, –	'*'*
	*Pst*S2,v27	–, 2, –, –, –, 6, 7, 8, 9, –, –, –, –, 25, 27, –, –, AvS	East and North Africa, West Asia
	*Pst*2,v1,v27	1, 2, –, –, –, 6, 7, 8, 9, –, –, –, –, 25, 27, –, –, AvS	East Africa, West Asia
	*Pst*S2,v3,v27	–, 2, 3, –, –, 6, 7, 8, 9, –, –, –, –, 25, 27, –, –, AvS	East Africa
	*Pst*S2,v10	–, 2, –, –, –, 6, 7, 8, 9, 10, –, –, 24, 25, –, –, –, AvS	East Africa, West Asia
	*Pst*S2,v10,v27	–, 2, –, –, –, 6, 7, 8, 9, 10, –, –, 24, 25, 27, –, –, AvS	West Asia
	*Pst*S2,v3,v10,v27	–, 2, 3, –, –, 6, 7, 8, 9, 10, –, –, 24, 25, 27, –, –, AvS	East Africa
	*Pst*S2_R1	1, 2, –, –, –, 6, 7, 8, 9, –, –, –, –, 25, 27, 32, Sp, AvS	Russia, Northwestern Region
	*Pst*S2_R2	1, 2, 3, 4, –, –, 6, 7, 8, 9, –, –, –, –, –, 27, 32, Sp, AvS	Russia, Northwestern Region

*Avirulence (–)/virulence characterization was performed using the following set of testers: *Yr1*, *Yr2*, *Yr3*, *Yr4*, *Yr5*, *Yr6*, *Yr7*, *Yr8*, *Yr9*, *Yr10*, *Yr15*, *Yr17*, *Yr24*, *Yr25*, *Yr27*, *Yr32*, Spalding Prolific (Sp), Avocet S (AvS).

## CONCLUSIONS

The molecular genetic analysis of populations of the wheat yellow rust pathogen for the presence of highly aggressive groups of races *PstS*1 and *Pst*S2 was performed in Russia for the first time. Isolates belonging to the PstS2 group have been found in the samples of the Northwestern population collected in the Leningrad Oblast. Isolates of the invasive group demonstrate two phenotypes of virulence different from those identified in other countries. The first identification of invasive races in the territory of Northwestern Russia indicates the need for the annual monitoring of regional populations of *P. striiformis.*
