# Erratum to: **Highly Aggressive Invasive Race Group** ***Pst*S2 in Russian Populations of the Wheat Yellow Rust Pathogen**

**DOI:** 10.1134/S0012496623050071

**Published:** 2024-01-25

**Authors:** E. L. Shaydayuk, E. I. Gultyaeva

**Affiliations:** All-Russian Institute of Plant Protection, 196608 St. Petersburg, Russia

The article “Highly Aggressive Invasive Race Group *Pst*S2 in Russian Populations of the Wheat Yellow Rust Pathogen,” written by E.L. Shaydayuk and E.I. Gultyaeva, was originally published electronically in Springer-Link on October 13, 2023 without Open Access. After publication in volume 511, pages 235–240, the authors decided to make the article an Open Access publication. Therefore, the copyright of the article has been changed to © The Author(s) 2023 and the article is forthwith distributed under the terms of a Creative Commons Attribution 4.0 International License (http://creativecommons.org/licenses/by/4.0/, CC BY), which permits use, duplication, adaptation, distribution and reproduction of a work in any medium or format, as long as you cite the original author(s) and publication source, provide a link to the Creative Commons license, and indicate if changes were made.

